# Global, regional and national burden of skin cancer from 1990 to 2021: Analysis of risk factors and prediction of trends in 2035

**DOI:** 10.1097/MD.0000000000049407

**Published:** 2026-07-03

**Authors:** Haoxuan Sun, Jun Fu, Jingjing Li, Wei Yuan, Jiangbo Xin

**Affiliations:** a Department of Oral and Maxillofacial Surgery, Hebei Eye Hospital, Xingtai, China.

**Keywords:** ARIMA model, Global Burden of Disease, public health, skin cancer, socio-demographic index

## Abstract

Skin cancer is among the most common malignancies worldwide, mainly classified into malignant skin melanoma (MSM) and nonmelanoma skin cancer (NMSC). This study aimed to comprehensively analyze the global burden and temporal trends of skin cancer, focusing on the incidence, prevalence, mortality, and disability-adjusted life years (DALYs) from 1990 to 2021. Data on incidence, mortality, and disability-adjusted life years (DALYs) for skin cancer were obtained from the Global Burden of Disease 2021 database across 204 countries and territories. Both crude numbers and age-standardized rates (ASIR, ASMR, and ASDR) were analyzed. Temporal trends were evaluated using estimated annual percentage change, and autoregressive integrated moving average (ARIMA) models were applied to project age-standardized rates, rather than crude counts, through 2035. In 2021, there were 3,03,105 MSM cases, 18,99,907 squamous cell carcinoma cases, and 44,36,939 basal cell carcinoma cases globally. From 1990 to 2021, the global ASIR of MSM increased by 19.32% (95% UI: 13.60–23.62%), squamous cell carcinoma by 67.34% (95% UI: 42.13–95.42%), and basal cell carcinoma by 63.30% (95% UI: 52.54–77.53%). High-SDI regions showed the highest incidence levels, whereas middle-SDI regions bore relatively higher mortality and DALY burdens. Males consistently exhibited higher incidence, mortality, and DALYs than females, and the disease burden increased markedly with age, particularly among individuals aged ≥ 70 years. ARIMA projections suggest subtype-specific trends, with age-standardized incidence and DALY rates remaining stable or declining for most skin cancer subtypes through 2035. Significant regional disparities persist in the burden of skin cancer. While high-income regions report higher incidence, low-income regions face greater challenges in early detection and treatment. Strengthening UV protection awareness, promoting early screening, and improving healthcare access are essential to reduce the future global burden of skin cancer.

## 1. Introduction

Skin cancer is one of the most common malignancies worldwide, with its incidence and disease burden steadily rising globally due to population aging and environmental changes.^[1]^ According to the Global Burden of Disease (GBD) study, skin cancer is primarily categorized into malignant skin melanoma (MSM) and nonmelanoma skin cancer (NMSC), with NMSC mainly consisting of basal cell carcinoma (BCC) and squamous cell carcinoma (SCC).^[2,3]^ On the other hand, NMSC, despite having a lower fatality rate, is a major contributor to the global burden of skin cancer due to its high incidence.^[4]^ Multiple factors, including population aging, increased ultraviolet radiation (UVR) exposure, immunosuppressive therapy, and advancements in diagnostic technologies, influence the incidence and burden of skin cancer.^[5,6]^

The GBD framework provides a comprehensive platform to quantify disease burden using standardized indicators including incidence, prevalence, mortality, and disability-adjusted life years (DALYs).^[7,8]^ However, despite increasing research on skin cancer, a comprehensive and updated global assessment incorporating the latest GBD 2021 data remains limited. Moreover, understanding future trends is essential for healthcare planning and policy formulation. Compared with simple extrapolation approaches, autoregressive integrated moving average (ARIMA) models incorporate temporal autocorrelation and can provide more robust short- and medium-term forecasts of disease burden.^[9,10]^

Therefore, this study aimed to: describe the global, regional, and national burden of MSM and NMSC from 1990 to 2021 using GBD 2021 estimates; analyze temporal trends across different socio-demographic index (SDI) regions; and forecast future age-standardized incidence and DALY trends through 2035 using an ARIMA model. These findings are expected to provide evidence to support targeted prevention, early detection, and resource allocation strategies.

## 2. Materials and methods

This study systematically analyzed the global burden of skin cancer, including MSM and nonmelanoma skin cancer (NMSC), using data from the GBD study from 1990 to 2021. The analysis includes incidence, mortality, and DALYs, and uses various statistical methods to predict the global burden trend of skin cancer for the next 14 years (2022–2035). The methodology is outlined as follows:

### 2.1. Data source and study population

The data for this study were derived from the GBD 2021 database, covering cross-sectional data from 204 countries and regions worldwide from 1990 to 2021. Data include indicators such as the numbers of cases and deaths, stratified by gender, age, socio-demographic index (SDI), and geographic region. This study utilized publicly available, de-identified data, and as such, ethical approval was not required.

This study used de-identified, publicly available, aggregated population estimates from the GBD 2021 database, with no access to individual-level data. According to international guidance on secondary analyses of anonymized data, institutional review board approval and patient consent were not required. All primary data sources included in the GBD framework obtained the necessary ethical approvals before contributing data.

### 2.2. Definition of disease

In this study, skin cancer is classified into two main types – MSM: refers to malignant tumors originating from melanocytes in the skin, which are typically treatable through surgery in the early stages but have high metastatic potential and lethality in advanced stages. Nonmelanoma skin cancer (NMSC): refers to all skin cancers other than melanoma, with basal cell carcinoma (BCC) and squamous cell carcinoma (SCC) being the most common types. BCC is usually locally invasive with a low metastatic rate, whereas SCC has a higher rate of metastasis and invasiveness. BCC and SCC share a lineage with keratinocytes and are often collectively referred to as keratinocyte carcinoma (KC).

### 2.3. Data analysis and burden evaluation

Burden indicators: this study used multiple disease burden indicators, including incidence, mortality, and DALYs. Both incidence and mortality were presented using ASRs (age-standardized incidence rate [ASIR] and age-standardized mortality rate [ASMR]), while DALYs were calculated based on incidence, mortality, and quality of life during illness.

Model selection and data integration: the study used the DisMod-MR 2.1 (Disease Modeling, Bayesian Meta-Regression) model to integrate incidence and mortality data from different countries and regions. The DisMod-MR 2.1 model, based on Bayesian statistics, is capable of handling cross-sectional and longitudinal data from various sources and time periods, ensuring internal consistency between data. Input data included national and regional cancer registry data, survey data, literature reports, and related clinical research data.

Disease definition and data standardization: different subtypes of skin cancer were classified according to the International Classification of Diseases (ICD-10). ASRs (ASIR and ASMR) were adjusted using the GBD World Standard Population to facilitate comparisons across different regions and periods.

### 2.4. Statistical analysis methods

Trend analysis: linear regression models were used to assess the estimated annual percentage change in the incidence, DALYs, and mortality of skin cancer globally and in different regions from 1990 to 2021. Estimated annual percentage change was used to quantify trends in burden indicators over specific periods. The formula for the model is:


$$EAPC=\left(e^{\beta }-1\right)\times 100\%,$$

where β is the slope of the regression model (global trends in the burden of nonmelanoma skin cancer).^[11,12]^ To characterize temporal patterns, we analyzed the entire continuous period from 1990 to 2021 rather than dividing the timeline into arbitrary subperiods, in order to make full use of all available annual estimates and ensure temporal consistency.

Time series analysis and forecast model: based on data from 1990 to 2021, the study used the autoregressive integrated moving average (ARIMA) model to predict ASIR and age-standardized DALY rates (ASDR) from 2022 to 2035. Auto-ARIMA was applied to iteratively evaluate multiple model specifications and select the optimal combination of parameters (p, d, q) based on information criteria, including the Akaike Information Criterion and Bayesian Information Criterion. Detailed model selection results are provided in Table S1, Supplemental Digital Content 1.

### 2.5. Uncertainty analysis

To assess the uncertainty of the estimates, 95% uncertainty intervals (UIs) were calculated using 1000 posterior draws. For historical estimates (1990–2021), the UIs reflect the inherent uncertainty of the GBD 2021 modeling framework and were derived directly from the posterior simulations provided by IHME. For future projections (2022–2035), additional uncertainty arising from time series forecasting was incorporated by simulating predictive distributions from the fitted ARIMA models, and the 2.5th and 97.5th percentiles of the simulated series were taken as the 95% UIs. The expanding uncertainty ranges in long-term projections therefore represent the accumulation of statistical forecasting uncertainty rather than different hypothetical policy or exposure scenarios.

### 2.6. Data visualization and presentation of results

Data analysis and visualization were performed using R software (Version 4.3.3; R Foundation for Statistical Computing). The study results were presented in various chart forms (such as line charts, scatter plots, and heat maps) to illustrate trends in the incidence, mortality, and DALYs of skin cancer across different countries and regions, providing a more intuitive representation of the global characteristics of skin cancer burden.

## 3. Results

### 3.1. Current global burden of skin cancer (2021)

In 2021, the number of MSM cases globally across all genders and age groups was 3,03,104.61 (95% UI: 2,81,717.64–3,18,904.82). The number of squamous cell carcinoma (SCC) cases was 18,99,907.05 (95% UI: 16,88,002.74–21,50,029.57), and the number of basal cell carcinoma (BCC) cases was 44,36,939.04 (95% UI: 39,07,156.85–49,55,955.19). The incidence, mortality, and age-standardized rates (ASR) of all 3 types of skin cancer were higher in males than in females.

In 2021, the incidence of MSM (2,04,511.18 cases, 95% UI: 1,91,890.97–2,12,086.9), DALYs (7,25,831.64, 95% UI: 6,82,352.45–7,69,618.4), and mortality (28,891.87 cases, 95% UI: 26,531.41–30,286.67) were all highest in high SDI regions. The lowest incidence, DALYs, and mortality of MSM were found in low SDI regions, while the lowest ASR was found in low-middle SDI regions. For SCC, both the incidence and ASR were also highest in high SDI regions, whereas DALYs, mortality, and their ASR were highest in middle SDI regions. The lowest incidence, DALYs, and mortality, along with their ASR, were all observed in low SDI regions. The incidence and DALYs of BCC and their ASR were highest in high SDI regions, while the lowest values for these metrics were observed in low SDI regions (Table 1).

**Table 1:** Global incidence, disability-adjusted life years (DALYs), and mortality of skin cancer in 2021 and corresponding changes from 1990 to 2021.

	2021		2021		2021		EAPC 1990–2021		
	Incidence	ASR	DALYs	ASR	Death	ASR	ASIR	ASDR	ASMR
Malignant skin melanoma									
Both	3,03,104.61 (2,81,717.64–3,18,904.82)	3.56 (3.31–3.75)	16,78,836.31 (14,74,533.66–18,37,368.79)	19.63 (17.25–21.5)	61,549.73 (54,852.45–66,265.02)	0.73 (0.65–0.79)	0.65% (0.32–0.98%)	−0.67% (−0.82 to −0.52%)	−0.43% (−0.58 to −0.29%)
Female	1,41,788.75 (1,30,334.77–1,53,059.96)	3.16 (2.9–3.41)	7,36,431.92 (6,15,594.67–8,51,312.29)	16.55 (13.77–19.21)	27,227.73 (23,212.24–30,950.45)	0.59 (0.51–0.68)	0.41% (0.12–0.70%)	−0.81% (−0.92 to −0.69%)	−0.67% (−0.78 to −0.57%)
Male	1,61,315.86 (1,50,889.89–1,71,042.66)	4.1 (3.82–4.36)	9,42,404.39 (8,22,097.6–10,56,864.5)	23.23 (20.33–26)	34,322 (30,494.36–37,624.84)	0.9 (0.81–0.99)	0.89% (0.52–1.25%)	−0.54% (−0.72 to −0.36%)	−0.21% (−0.39 to −0.03%)
Nonmelanoma skin cancer (squamous cell carcinoma)									
Both	18,99,907.05 (16,88,002.74–21,50,029.57)	22.38 (19.9–25.27)	12,10,874.53 (10,68,480.71–13,34,385.86)	14.31 (12.65–15.78)	56,913.23 (48,761.35–63,037.42)	0.69 (0.59–0.77)	2.06% (1.58–2.54%)	0.24% (0.1–0.31%)	0.31% (0.25–0.38%)
Female	7,12,429.91 (6,36,435.22–8,00,222)	15.3 (13.67–17.17)	4,94,195.89 (4,32,594.73–5,50,339.88)	10.8 (9.47–12.02)	24,725.28 (20,539.14–27,617.27)	0.53 (0.44–0.59)	1.83% (1.33–2.34%)	0.18% (0.10–0.27%)	0.19% (0.13–0.25%)
Male	11,87,477.14 (10,51,961.99–13,48,915.21)	31.53 (27.96–35.7)	7,16,678.64 (5,94,142.43–8,07,397.25)	18.58 (15.55–20.84)	32,187.95 (26,973.37–36,792.06)	0.92 (0.77–1.05)	2.10% (1.64–2.57%)	0.26% (0.18–0.35%)	0.39% (0.31–0.46%)
Nonmelanoma skin cancer (basal cell carcinoma)									
Both	44,36,939.04 (39,07,156.85–49,55,955.19)	51.71 (45.7–57.58)	1997.85 (920.96–3770.95)	0.02 (0.01–0.04)	NA	NA	2.01% (1.58–2.44%)	1.64% (1.27–2.01%)	NA
Female	19,28,098.55 (16,91,674.69–21,59,949.8)	42.03 (36.89–47)	912.4 (418.2–1745.46)	0.02 (0.01–0.04)	NA	NA	1.59% (1.20–1.98%)	1.22% (0.90–1.53%)	NA
Male	25,08,840.49 (22,16,962.69–27,91,803.82)	64.29 (57.09–71.37)	1085.45 (500.34–2037.81)	0.03 (0.01–0.05)	NA	NA	2.32% (1.84–2.81%)	2.00% (1.57–2.44%)	NA

Values in parentheses represent 95% uncertainty intervals.

ASDR = age-standardized disability-adjusted life year (DALY) rate, ASIR = age-standardized incidence rate, ASMR = age-standardized mortality rate, ASR = age-standardized rate (per 1,00,000 population), DALY = disability-adjusted life year, EAPC = estimated annual percentage change.

**Figure 1.**
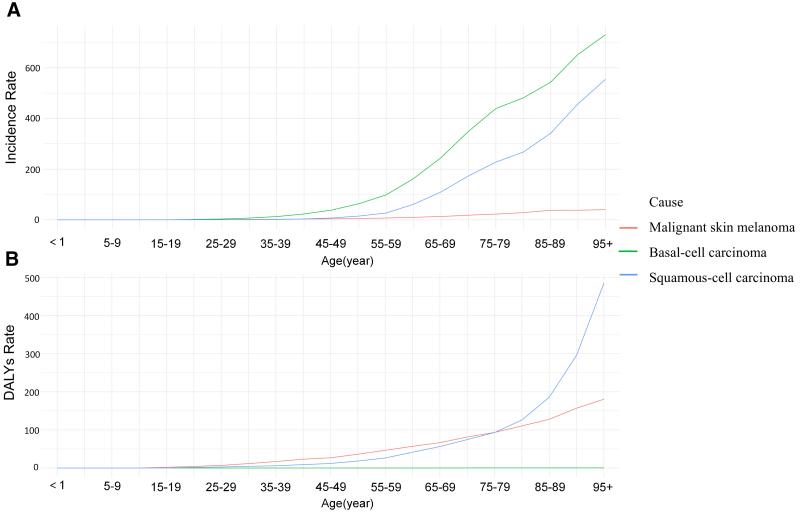
Global trends of 3 types of skin cancer across all age groups for both sexes in 2021. (A) Incidence, (B) DALYs. DALY = disability-adjusted life year.

Based on the global distribution of skin cancer across age groups in 2021, as shown in Figure 1, MSM begins to appear from age 15, while SCC and BCC appear from age 20. The incidence rates of all 3 types of skin cancer increase with age. BCC consistently exhibits a higher incidence rate than SCC, whereas MSM maintains a comparatively low incidence rate throughout. The DALY for MSM and SCC also increases with age. For individuals aged 15 to 75, the DALY rate for MSM surpasses that of SCC. However, from age 80 onward, the DALY rate for SCC rises sharply, exceeding that of MSM. The DALY rate for BCC remains consistently low across all age groups.

Between 1990 and 2021, globally, the countries with the largest percentage increase in ASIR of MSM were Mauritius (928.69%, 95% UI: 841.94–1002.46%), Cabo Verde (487.83%, 95% UI: 18.87–1103.68%), and Belarus (302.88%, 95% UI: 217.38–408.18%). The countries with the largest percentage decreases in ASIR were Kyrgyzstan (−34.15%, 95% UI: −51.04 to −13.27%), Turkmenistan (−22.33%, 95% UI: −43.68 to 5.56%), and Burundi (−21.55%, 95% UI: −54.24 to 40.65%).

Between 1990 and 2021, globally, the countries with the largest percentage increase in ASIR of squamous cell carcinoma (SCC) were China (565.84%, 95% UI: 512.44–627.24%), Portugal (133.58%, 95% UI: 98.65–185.11%), and the United States of America (130.23%, 95% UI: 88.38–176.71%). The countries with the largest percentage decreases in ASIR were Peru (−63.4%, 95% UI: −70.19 to −56.18%), Costa Rica (−58.37%, 95% UI: −66.6 to −50.52%), and Panama (−55.4%, 95% UI: −64.58 to −43.49%).

**Figure 2.**
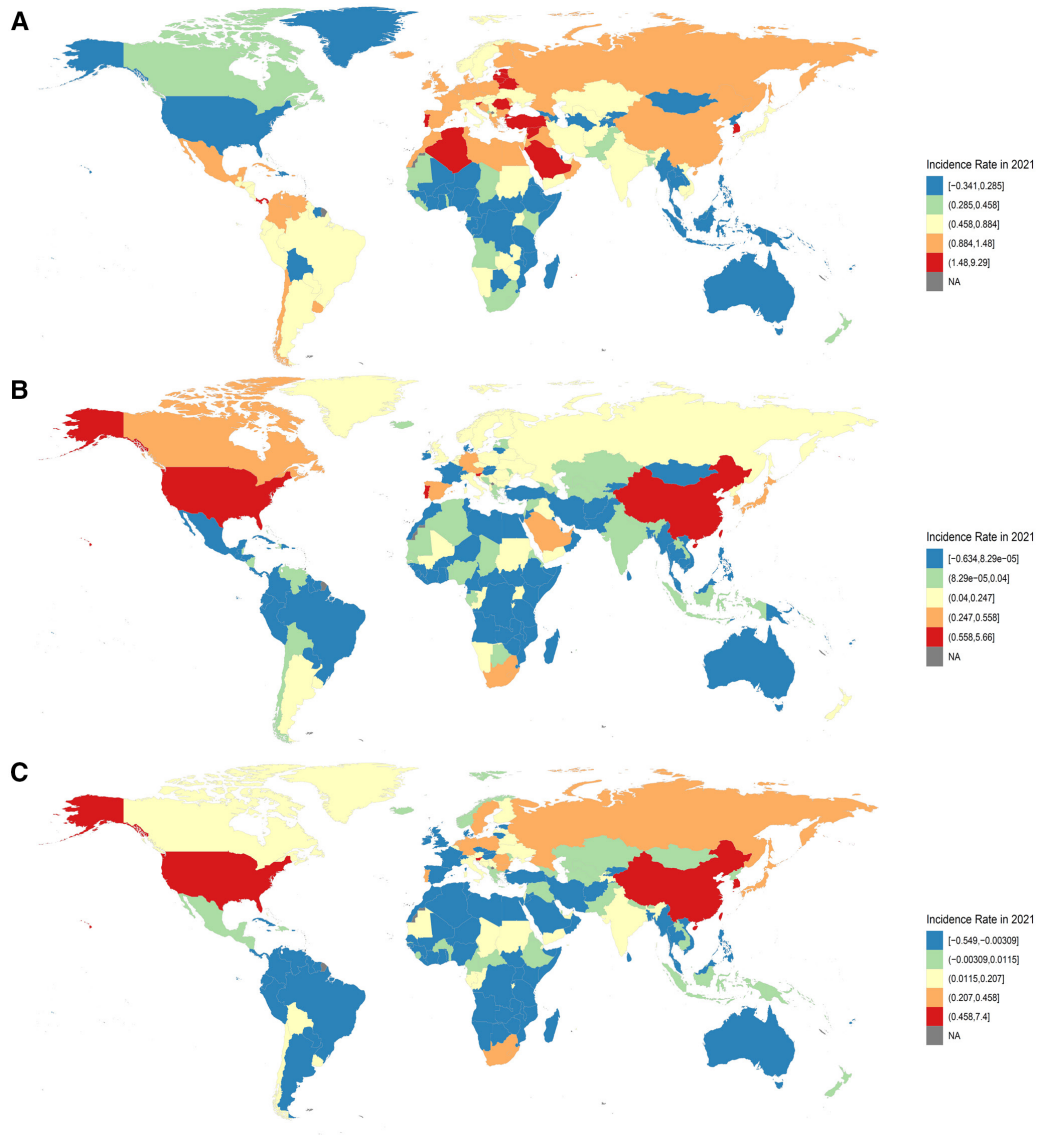
Changes in incidence rates of 3 types of skin cancer across countries and regions globally from 1990 to 2021. (A) MSM, (B) SCC, (C) BCC. BCC = basal cell carcinoma, MSM = malignant skin melanoma, SCC = squamous cell carcinoma.

Between 1990 and 2021, globally, the countries with the largest percentage increase in ASIR of basal cell carcinoma (BCC) were China (740.31%, 95% UI: 691.18–788.58%), the United States of America (131.5%, 95% UI: 107.06–170.91%), and the Republic of Korea (66.93%, 95% UI: 57.3–76.9%). The countries with the largest percentage decreases in ASIR were Taiwan (Province of China; −95.15%, 95% UI: −97.12 to −92.15%), Thailand (−54.92%, 95% UI: −63.88 to −46.72%), and Viet Nam (−42.79%, 95% UI: −53.8 to −29.93%; Fig. 2).

### 3.2. Temporal trends and projections of the global skin cancer burden (1990–2035)

Global trends: From 1990 to 2021, the incidence of all 3 types of skin cancer showed an increasing trend. The ASIR of MSM changed by 19.32% (95% UI: 13.6–23.62%), while that of SCC increased by 67.34% (95% UI: 42.13–95.42%) and BCC increased by 63.3% (95% UI: 52.54–77.53%). Globally, age-standardized DALYs and mortality rates for SCC and BCC showed an increasing trend, whereas MSM showed a declining trend (Table 1).

**Figure 3.**
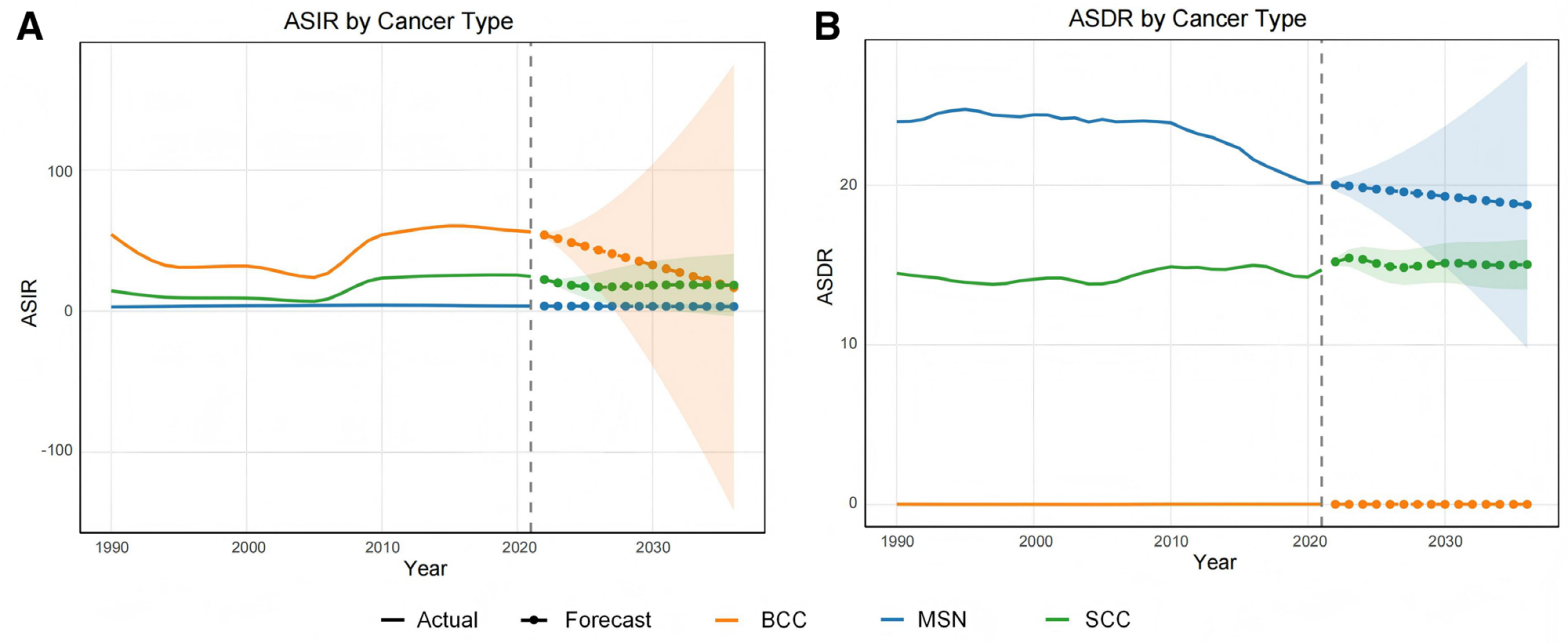
ARIMA-based projections of age-standardized rates of skin cancer from 2022 to 2035. (A) Age-standardized incidence rate (ASIR). (B) Age-standardized DALY rate (ASDR). ARIMA = autoregressive integrated moving average, ASDR = age-standardized disability-adjusted life year rate, ASIR = age-standardized incidence rate.

As shown in Figure 3A, ARIMA forecasts for 2022 to 2035 indicate divergent patterns in the ASIR across skin cancer subtypes. BCC is projected to decline, whereas SCC is expected to remain overall stable, and MSM is predicted to stay at a low and relatively stable level during the forecast period. In Figure 3B, projections of the age-standardized DALY rate (ASDR) suggest that MSM will continue to decrease gradually from 2022 to 2035. SCC is anticipated to remain largely stable or show a slight decline, while BCC is expected to remain at a very low and nearly unchanged level over the same period.

### 3.3. Analysis of factors affecting the burden of skin cancer

Environmental exposure: ultraviolet radiation (UVR) is a major environmental factor for skin cancer. Studies indicate that the level of UVR exposure is significantly correlated with skin cancer incidence, particularly in mid- to high-latitude regions (such as Australia, North America, and Europe). With climate change and ozone layer depletion, global UVR exposure levels are likely to increase in the future, which may heighten the risk of skin cancer.

Population aging: population aging is one of the main drivers of the increasing burden of skin cancer globally. As the sensitivity of the elderly population to UVR increases, the burden of skin cancer in those aged 70 years and older is expected to rise significantly.

Socioeconomic factors: national income, urbanization level, and accessibility to healthcare resources are all significantly related to the burden of skin cancer. High-income countries, due to better healthcare quality and disease management capacity, have relatively low age-standardized mortality rates for skin cancer, although their incidence rates remain high.

## 4. Discussion

### 4.1. Overall trends and epidemiological characteristics of global skin cancer burden

Based on the GBD database, this study systematically evaluated the epidemiological characteristics and trends in the burden of skin cancer (including malignant skin melanoma and nonmelanoma skin cancer) worldwide from 1990 to 2021. The results showed that the overall global burden of skin cancer has increased significantly over the past 30 years, especially the incidence and DALYs of nonmelanoma skin cancer (NMSC), which has become more pronounced in countries and regions with severe aging populations. Although NMSC has a relatively low fatality rate globally, its high incidence and significant impact on patient’s quality of life make it one of the major public health concerns worldwide.^[13]^

MSM, although accounting for only 2% of all skin cancer cases, has a high fatality and metastasis rate, making it a leading cause of skin cancer-related deaths. The age-standardized mortality rate (ASMR) of MSM has slightly decreased between 1990 and 2021, but due to an increasing incidence rate and an aging global population, the number of deaths and DALYs associated with MSM has still risen significantly.

### 4.2. Factors influencing skin cancer burden and regional differences

The study demonstrated significant regional differences in the incidence and burden of different types of skin cancer, closely related to environmental exposure, socioeconomic development, and health resource distribution. High-income countries (such as North America, Australia, and parts of Europe) have significantly higher skin cancer incidence rates than lower-income countries, partly due to higher ultraviolet radiation (UVR) exposure. Over the past few decades, these regions have implemented several prevention and screening measures for skin cancer, such as promoting sunscreen use, conducting public health education, and establishing screening programs, thereby achieving some success in reducing skin cancer mortality.^[14-16]^ However, despite the decline in mortality, the incidence and DALYs of skin cancer, especially the ASIR of MSM, have continued to rise over the past 32 years.

Conversely, there has been a notable increase in the burden of skin cancer in lower-middle and low-income countries (such as Sub-Saharan Africa and South Asia) in recent years. This phenomenon may be attributed to several factors: first, the lack of adequate resources for skin cancer prevention and early screening in these countries leads to delays in disease detection and diagnosis. Second, there is generally insufficient health education and public awareness, resulting in a lower prevalence of sun protection measures. Lastly, increased UVR exposure due to global climate change may further drive up skin cancer incidence in these regions.

### 4.3. Gender and age differences

The gender and age distribution characteristics of global skin cancer reveal disparities in the burden among different populations. The results showed that the incidence and mortality rates of skin cancer were significantly higher in males than in females. This higher burden among males may be associated with more frequent outdoor activities and less use of skin protection measures.

Age is another important factor influencing the burden of skin cancer. Globally, the incidence and mortality of skin cancer increase with age, with those aged 70 and older bearing the highest burden. Due to prolonged UV exposure and declining immune function with age, the elderly are at higher risk of developing skin cancer. As the global population continues to age, the burden of skin cancer among older adults is expected to rise further, becoming one of the main drivers of the increased global skin cancer burden.

### 4.4. Predictions and challenges for the future global skin cancer burden

Based on annual estimates from 1990 to 2021, our ARIMA forecasts indicate subtype-specific trajectories in the age-standardized burden of skin cancer from 2022 to 2035. In terms of incidence (ASIR), BCC is projected to decline, whereas SCC is expected to remain overall stable, and MSM is predicted to stay at a low and relatively stable level. For disease burden (ASDR), the model suggests a continued gradual decrease in MSM, while SCC is likely to remain broadly stable or decline slightly, and BCC is expected to persist at a very low and nearly unchanged level. These projected patterns imply that, although ASRs for some subtypes may stabilize or decrease, the overall health-system impact may remain substantial.

### 4.5. The main drivers of the rising global burden of skin cancer in the future include

Population aging: the elderly are at high risk for skin cancer, and with the global population aging, the burden of skin cancer in those aged 70 and older will significantly increase.

Climate change: the level of UVR exposure is significantly associated with skin cancer incidence. With the worsening of global climate change, ozone depletion, and increased UVR exposure, the incidence of skin cancer may continue to rise.

Health awareness and public health interventions: in lower-income countries, the lack of effective screening and prevention measures makes early detection and treatment of skin cancer difficult. As a result, these regions may face a higher burden in the future.

### 4.6. Recommendations for public health strategies and interventions

To address the rising global burden of skin cancer, this study proposes the following public health strategies and intervention recommendations:

Strengthen global collaboration on skin cancer prevention: countries should strengthen international collaboration and data sharing on skin cancer prevention and promote the formulation and implementation of global prevention strategies. In particular, there is an urgent need to introduce effective screening and early intervention measures in lower-income countries to reduce health inequalities related to skin cancer.

Promote skin cancer protection education and awareness: countries should raise public awareness of skin cancer and UV protection through health education and promotional campaigns, particularly targeting men and the elderly. Measures such as promoting sunscreen use, reducing sun exposure, and wearing protective clothing should be advocated to reduce the risk of skin cancer.

Establish screening and early intervention systems for high-risk populations: establish a screening and early diagnosis system focusing on the elderly and populations with prolonged UV exposure. Emerging diagnostic technologies (e.g., AI-assisted screening) and imaging techniques (e.g., dermoscopy) should be used to improve early detection and treatment outcomes for skin cancer.

Optimize health resource allocation and policy support: in regions with a heavy burden of skin cancer (such as North America and Australia), governments should increase investment in skin cancer research and prevention, optimize health resource allocation, and prioritize skin cancer prevention in public health agendas. For lower-income countries, financial and technical support should be provided to enhance their skin cancer prevention capacity.

### 4.7. Limitations and future research directions

This study mainly relied on GBD database data on the global burden of skin cancer, which may have limitations due to incomplete data and variations in data quality across regions. Additionally, differences in skin cancer diagnosis and treatment among countries and regions may affect the generalizability of the results. The GBD framework groups heterogeneous histological subtypes under broad categories, which may mask subtype-specific epidemiological characteristics and clinical relevance. Future research should further improve data collection in low-income countries and regions, establish a globally unified system for assessing the burden of skin cancer, and explore the complex relationships between skin cancer burden and environmental exposure and socioeconomic indicators. In addition, although UV exposure and environmental changes are discussed as potential contributors to future increases in skin cancer burden, our analysis did not incorporate region-specific quantitative UV trend data, and these interpretations are therefore based on existing literature rather than direct estimates from this study.^[17]^

In summary, the global burden of skin cancer has increased significantly over the past 30 years and is expected to continue rising in the coming years. Strengthening global collaboration on skin cancer prevention, promoting health education, and enhancing early screening and diagnostic capabilities are expected to effectively reduce the impact of skin cancer on global health and alleviate the associated burden.

## Acknowledgments

We thank all the individuals who contributed to the Global Burden of Disease Study 2021 for their extensive support in finding, cataloguing, and analyzing data and facilitating communication between and among team members.

## Author contributions

**Formal analysis:** Jingjing Li.

**Supervision:** Jun Fu, Wei Yuan.

**Validation:** Jingjing Li.

**Visualization:** Jun Fu.

**Writing – original draft:** Haoxuan Sun.

**Writing – review & editing:** Jiangbo Xin.


