# Supplementary material for: Global, regional and national burden of skin cancer from 1990 to 2021: Analysis of risk factors and prediction of trends in 2035
Source: Medicine (Baltimore). 2026 Jul 3;105(27):e49407. doi: 10.1097/MD.0000000000049407 (PMC13337087; doi:10.1097/MD.0000000000049407)
Supplement: Supplementary file 1 [file medi-105-e49407-s001.docx]

| Subtype | Outcome | Selected ARIMA (p,d,q) | AIC | BIC | Ljung–Box p value |
| --- | --- | --- | --- | --- | --- |
| MSM | ASIR | ARIMA(1,2,0) | -100.19 | -97.39 | 0.2734 |
| BCC | ASIR | ARIMA(0,2,2) | 78.79 | 82.99 | 0.5624 |
| SCC | ASIR | ARIMA(2,1,1) | 45.15 | 50.89 | 0.6831 |
| MSM | ASDR | ARIMA(1,2,0) | -12.86 | -10.06 | 0.7898 |
| BCC | ASDR | ARIMA(2,1,1) | -394.96 | -389.22 | 0.6596 |
| SCC | ASDR | ARIMA(2,1,0) | -38.44 | -34.14 | 0.6922 |

Supplement Table 1. Selected ARIMA (p,d,q) models and residual diagnostics for forecasting ASIR and ASDR by skin cancer subtype.

Abbreviations: ARIMA, autoregressive integrated moving average; AIC, Akaike information criterion; BIC, Bayesian information criterion; ASIR, age-standardized incidence rate; ASDR, age-standardized disability-adjusted life year (DALY) rate; MSM, malignant skin melanoma; BCC, basal cell carcinoma; SCC, squamous cell carcinoma.
